# Implementation of Virtual Reality Pain Alleviation Therapeutic into Routine Pediatric Clinical Care: Experiences and Perspectives of Stakeholders

**DOI:** 10.1089/jmxr.2024.0018

**Published:** 2024-09-26

**Authors:** Helen Girin, Megan Armstrong, Kim A. Bjorklund, Christopher Murphy, Julie B. Samora, Jonathan Chang, Daniel J. Scherzer, Henry Xiang

**Affiliations:** ^1^ Center for Pediatric Trauma Research, The Abigail Wexner Research Institute, Nationwide Children’s Hospital, Columbus, Ohio, USA.; ^2^ Center for Injury Research & Policy, The Abigail Wexner Research Institute, Nationwide Children’s Hospital, Columbus, Ohio, USA.; ^3^ Section of Plastic Surgery, Nationwide Children’s Hospital, Columbus, Ohio, USA.; ^4^ Department of Plastic Surgery, The Ohio State University College of Medicine, Columbus, Ohio, USA.; ^5^ Department of Orthopedics, Nationwide Children’s Hospital, Columbus, Ohio, USA.; ^6^ Division of Emergency Medicine, Nationwide Children’s Hospital, Columbus, Ohio, USA.; ^7^ Department of Pediatrics, The Ohio State University College of Medicine, Columbus, Ohio, USA.

**Keywords:** pediatric, virtual reality, acute pain, anxiety, implementation

## Abstract

Virtual reality (VR) needs to be implemented in clinical settings to improve pediatric patient care during painful medical procedures. Engaging doctors and nurses to lead the adoption of new clinical techniques can facilitate the transition from research to routine practice. Integrating VR into routine clinical practice could reduce patient pain and anxiety, making medical procedures smoother and more efficient. This feasibility pilot quality improvement (QI) project provides evidence for broader expansion and implementation of VR into different clinical areas. Medical providers (doctors and nurses) implemented VR during brief pediatric medical procedures and completed a demographics and feasibility survey. Qualitative feedback from semi-structured interviews indicated VR’s ease of use and effectiveness in reducing anxiety and easing medical procedures. Patients (*n* = 30) played the VR game during either their medically necessary pin-pulling or needlestick procedures within three clinical environments. Children ranged from 5–16 years and were 50% male. The majority of patients reported enjoyment (mean 8.2 out of 10) with VR during the procedure, and only one minor technical issue was reported. Qualitative semi-structured interview data showed the benefits of using VR, including its ease of use, decreased observed anxiety, and patients having an easier time getting through the medical procedures. Feedback from medical providers regarding the dissemination of VR into pediatric clinical environments showed promising results. Standardized guidelines are needed to further implement VR pain alleviation into standard care in real-world clinical settings. This pilot study suggests that VR could be a valuable tool in pediatric pain management, warranting further research and broader clinical implementation.

## Introduction

Pain is an important component of clinical care and requires thoughtful pain management strategies to avoid psychological and emotional strain for patients and family members. Acute pain is “the physiological response and experience to noxious stimuli that can become pathological, is normally sudden in onset, time-limited, and motivates behaviors to avoid actual or potential tissue injuries.”^1^ Opioid medications are often used for acute pain management post-injury or during procedures, but the opioid crisis has led to efforts to reduce opioid prescriptions through state cap laws.^2^ The United States Department of Health and Human Services named the opioid epidemic in 2017 as a public health emergency and renewed this declaration yearly thereafter.^3^ Notably, in the pediatric population, it has been shown that opioid exposure post-surgery is associated with persistent opioid use among opioid-naïve patients.^4^ Non-pharmacological pain management methods, such as virtual reality (VR), are particularly relevant as they can address acute pain needs while sparing opioid side effects and the potential for misuse.

Various non-pharmacological methods like music, movies, and games have been employed in pediatric settings to distract patients from painful procedures, showing varying degrees of success.^5^ Previous studies have demonstrated VR’s effectiveness in reducing pain during pediatric procedures like needlesticks^6,7^ and burn dressing changes,^8,9^ highlighting its potential as a distraction tool. Our team developed a virtual reality pain alleviation therapeutic (VR-PAT) to address the procedural pain experienced during pediatric burn dressing changes in the outpatient clinic.^8^ This VR-PAT was a fun, child-appropriate VR game developed at our institution with feedback from patients and clinicians. It was also hosted on an iPhone and displayed on a low-cost VR headset. Considerations were made to ensure the system was lightweight and highly portable to ensure it was practical for a clinical setting. In our previous randomized controlled trial, participants using the active VR-PAT self-reported clinically meaningful lower pain scores than those who only received the standard of care.^8^ Nurses also reported that the VR-PAT was easy to implement in the clinic and found it to be helpful for the majority of dressing changes.^8^ Through further analysis, we found a link between anxiety and pain, with self-reported anxiety prior to the dressing change being significantly associated with self-reported pain following the procedure.^10^ Soumil et al. further discovered that the perceived realism, fun, and engagement of VR-PAT significantly impact the effectiveness of these interventions for reducing pain.^11^ Finally, our team found a clinically significant reduction in self-reported pain while using the VR-PAT during home care of burn injuries.^12^ None of our participants reported serious adverse events, which supported our hypothesis that VR-PAT is a safe non-pharmacological pain alleviation therapeutic.

While VR has been found safe and effective for acute pain alleviation during pediatric procedures, there are gaps in the dissemination and clinical implementation of these novel interventions outside the research domains. Researchers provided evidence that it can take decades to move evidence of effectiveness in the research domain to clinical implementation.^13^ Through research dissemination efforts, our research team was approached by clinicians to trial our VR-PAT in their clinical settings. This pilot feasibility project aimed to report our experience of having medical staff (doctors or nurses) in the emergency department (ED), Plastic Surgery Clinic, and Orthopedic Clinic implement VR-PAT with little or no help from the research team.

## Methods

### Study design and population

**FIG. 1.**
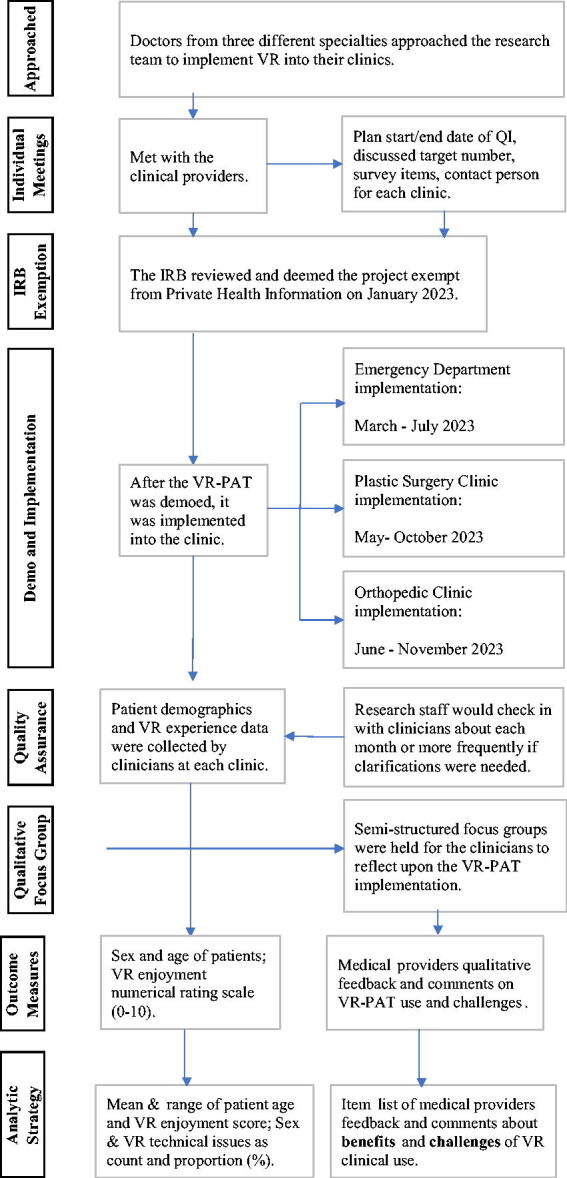
Flow chart of VR-PAT implementation and evaluation. VR-PAT, virtual reality pain alleviation therapeutic.

Multiple clinical providers at a large academic pediatric hospital approached the research team about the feasibility and implementation of using VR-PAT in their clinical settings (Fig. 1). We conducted separate meetings with each participating medical provider to align on the goals and expectations of the VR-PAT implementation. The whole team (researchers and medical providers) agreed upon an initial target sample size of 10 per clinic, and the data/information saturation assessed by the medical providers was used to guide the data collection and the length of the study. The Institutional Review Board (IRB) at our research institute reviewed this project and determined that the proposed activity was not research involving human subjects as defined by Department of Health and Human Services and Food and Drug Administration regulations. Following the IRB review, an in-person meeting was scheduled with each medical provider to demo the headset and answer any questions. Brief, de-identified data on the VR-PAT’s effectiveness and ease of use were collected via a paper survey completed by medical providers after each procedure. The research team did not have any clinical involvement with the implementation of the VR other than assisting with any questions or problems should they arise. Completed surveys were scanned and sent to a point person on the research team after each participant encounter or at the end of the month if prompted. Survey data were entered into a Research Electronic Data Capture^14,15^ database. After each clinic completed a convenience sample of 10 surveys (*n* = 30 total) and the medical providers thought a data/information saturation was reached, semi-structured interview meetings were scheduled to collect more qualitative data about the medical providers’ experience in implementing the VR during their medical practice. These interviews were recorded, and a researcher took notes on common themes.

Participants were children aged 5–17 years who spoke English, with no history of seizures or adverse reactions to VR. These were patients being treated for pin-pulling procedures, in which a medical provider will remove a pin that was inserted into a bone that had been previously fractured, in the Plastic Surgery and Orthopedic Clinics or needlesticks in the ED.

### VR game

Our published research used the smartphone version of the VR-PAT app;^8,11,12^ however, we have since transferred this app onto a head-mounted display (PICO Neo3 Pro Eye). Each clinic received a VR PICO headset pre-loaded with the Virtual River Cruise game, designed to immerse children in a calming virtual environment with minimal physical interference. The game gave the child a first-person view of floating gently down a river in a boat with a penguin companion. The child moved their eyes from side to side to see a winter landscape and crystals that would appear within the field of vision. Looking directly at the crystal allowed it to be broken open to receive points on the scoreboard and a lower temperature on the thermostat. The difficulty of breaking the crystals increased with the length of time spent playing the game. The Virtual River Cruise game was played by having children slightly tilt their heads (no hand, finger, or arm involvement), which minimized interference with the medical procedure. The enjoyability and immersive environment were designed to create a calming virtual environment to reduce anxiety. The game automatically started when the headset was powered on to limit additional steps for medical staff. It lasted indefinitely so that the VR-PAT could be played throughout the entire medical procedure with a fully charged device. Details of the smartphone-based VR-PAT have been described in previous publications.^8,11^

### Survey questions

The medical care providers (a doctor or nurse) filled out a survey for each participant (Supplementary Data S1). Survey items included: age of patient, sex, month and year of VR-PAT use, patient-reported VR-PAT enjoyment, and technical issues with using the VR.

Patient-reported VR-PAT enjoyment was obtained by asking, “On a scale of 0–10, how much did you like playing the VR-PAT game during your procedure?” (0 = did not like it at all, 10 = liked it the most possible). Upon completion of the medical procedure, the medical provider reported any technical issues with using the VR-PAT.

### Post-implementation semi-structured interviews

After each clinic had collected a total of 10 surveys, all medical providers thought that data/information saturation had been reached. Semi-structured interviews were conducted with medical providers post-implementation to gather qualitative feedback on VR-PAT use. Common themes were identified and noted by researchers. These individual interviews were meant to collect qualitative feedback about the clinic’s experience implementing the VR-PAT with their patients. The following questions were shared to capture the common themes: “Describe the experience of using the VR at your clinic,” “What do you think about the VR that worked well in the clinic setting?” “What do you think about the VR did not work well in the clinic setting?” “Do you have any suggestions for improvement, whether it be the survey or the VR game?” “In your opinion, what should we do to disseminate into other clinics or hospitals?”

### Statistical analysis

Mean and range were calculated for continuous variables (age and enjoyment score). Sex and technical issues are reported as count and proportion (%). All analyses were conducted using functions in Microsoft Excel 365. No formal content or thematic analyses of the qualitative interviews were conducted in this feasibility pilot study.

## Results

### VR-PAT clinical implementation

The study population was evenly split by gender (50% male) with a mean age of 9.8 years (range 5–16) (Table 1). Children from the ED were younger than children in the Orthopedic & Plastic Surgery clinics who needed pin-pulling (mean 7.8 and 10.9 years, respectively). Children across the study clinics reported enjoying the VR-PAT (mean 8.2, range 0–10). The pin-pulling cohort reported one technical problem in which the patient had problems popping the crystals. This was a minor issue that was resolved easily by restarting the headset. Some medical providers reported that they were prone to motion sickness and could not wear the VR-PAT and experience the game for themselves. Therefore, they had to rely on the researcher’s explanation of the game to guide the patient through the game.

**Table 1.** Clinical Implementation of VR-PAT for Quality Improvement

	Total *n* = 30	ED *n* = 10	Pin-Pulling *n* = 20
Age, Mean (range)	9.8 (5–16)	7.8 (5–14)	10.9 (6–16)
Male, *n* (%)	15 (50%)	6 (60%)	9 (45%)
Female, *n* (%)	15 (50%)	4 (40%)	11 (55%)
Enjoyment Score^*^, Mean (range)	8.2 (0–10)	7.5 (0–10)	8.7 (3–10)
Technical Issues, *n* (%)	1 (3%)	0 (0%)	1 (5%)

^*^ How much did you like playing the VR game during your procedure? (0 = did not like at all, 10 = liked it the most possible).

ED, emergency department; VR, virtual reality; VR-PAT, VR pain alleviation therapeutic.

### Qualitative outcomes

Table 2 reports common themes from the semi-structured interviews with medical providers. Clinics reported observing reduced anxiety among children using the VR-PAT, noting less need for parental reassurance and easier procedure completion.

**Table 2.** Benefits and Challenges of Using VR-PAT from Semi-Structured Interviews

Benefits	Challenges
Patient had an easier time with medical procedure	There are children who are too anxious to accept VR-PAT
Medical providers perceived less anxiety in patients	Needs more gamification/competition for older kids
Easy to use after explanation to medical staff	VR-PAT was designed for six-year-olds and above
Easy to introduce and explain the game to the patients	
Little time required to set up the headset/game	
Integrating it into the clinic flow was not difficult	
Can be played for the entirety of the medical procedure	
Minor technical difficulties could be resolved by medical staff	

VR-PAT, virtual reality pain alleviation therapeutic.

Specifically, a clinician doing pin-pulling procedures noted, “*VR used during pin-pulling provided the distraction technique for my patients that has so far eluded them. Being immersed in VR and less aware of the surroundings that are frequently anxiety-provoking (seeing the pin-pulling device, the pin itself in the skin, and blood) allowed patients to relax and made the whole experience much less traumatic. Many of the patients were barely aware of when the pins were removed and wanted to continue engaging with the VR for as long as possible, which is incredible.*”

They reflected upon children’s anxiety without VR-PAT with patients pulling away from the procedure and parent reassurance. Medical providers noted that when the child was fully immersed in the VR-PAT, they would remain still, and the parent would usually have minimal interaction with the child. However, some kids were too anxious to allow visual obstruction and needed to see what the medical staff were doing during the procedures. These patients were not able to be fully immersed in the VR environment.

The medical providers found it difficult to comment on observed pain, as these pin-pulling and needlestick procedures were very short but felt that the decreased anxiety might have helped with perceived pain as well.

The easy-to-use features of the VR-PAT allowed the medical providers to use it in their clinical setting without prolonging the procedure time. Having the Virtual River Cruise game start immediately when the VR headset was powered on and the game playing continuously allowed the child to be fully immersed into the game during the whole procedure. The game was also easy to introduce and explain to patients, so they did not have a steep learning curve. There was little technical assistance required from the research team after having a scheduled demonstration to the medical providers on how to use and implement the VR-PAT.

One ED physician commented, “*VR implementation in the pediatric emergency department was readily accepted by the vast majority of patients surveyed. Both the patients and their caregivers found the experience both satisfactory and helpful for anxiolysis. Both nursing and physician staff had no technical issues with implementation of the VR headset*.”

A challenge medical providers encountered was that the game seemed to work better for younger children, as older children expressed the desire for a more challenging or competitive game. Also, since the game was designed and tested in children aged 5–17 years, this limited the ability to recruit patients younger than 5 years old.

## Discussion

The implementation of VR-PAT in real-world clinical settings showed promising results, with significant reductions in patient anxiety and high enjoyment levels reported. While this project has a small convenience sample, we were able to provide evidence that this VR pain and anxiety intervention was easy to implement without impeding the required medical procedure. Children also reported enjoyment in playing the game, with clinicians noting an observed decrease in anxiety before and during the procedures.

Participants in this project were evenly distributed between sexes (50% female) and covered a range of ages (5–16 years). Only one technical issue was reported that was easy to resolve within the clinic without outside assistance. This is important for further clinical implementation of VR-PAT. Orthopedic clinics report continued use of VR-PAT six months after data collection of this pilot feasibility study ended. Most children reported that they enjoyed playing the game (8.2 out of a maximum 10). This enjoyment score ranged from 0 to 10, which is not unusual. However, medical providers noticed that a few patients in our prior VR research also expressed a desire to watch the procedure and did not like having their eyes covered. Additionally, the Virtual River Cruise game was designed for a broad age range and to support many skill levels. Because of this, some older patients and those with more video game experience could feel that the game is too easy and needs more gamification to create a more challenging experience. This clinical implementation is reflective of prior VR-PAT research in which most children report enjoying playing VR during medical procedures.^8,12^

During the semi-structured interviews, clinicians notably mentioned that patients had an easier time with the medical procedures. Clinicians talked about how without VR, patients were very anxious prior to the pin-pulling or needlestick procedures, and the anxiety often added time to the procedure with parents and medical staff offering encouragement. We did not include a metric to capture anxiety in our survey, but anecdotally, clinicians reported that when patients were fully immersed in the VR-PAT early in the appointment, they were able to complete the procedure more quickly and without additional reassurance. Other researchers have found similar results during pediatric medical procedures. These findings align with previous studies by Kilic et al. and Gold et al., which also reported reduced anxiety and improved patient experiences with VR interventions.^16,17^ The preliminary insights provided in the semi-structured interviews warrant more formal research into VR-PAT’s impact on procedural anxiety.

This project should be interpreted within the context of several limitations. This study’s small sample size and single-hospital setting limit the generalizability of the results. Future studies should include larger, more diverse populations and multiple institutions. Because of the study’s nature and its purpose, a formal implementation research framework such as the Consolidated Framework for Implementation Research^18^ was not applied. Furthermore, the survey forms were not administered anonymously. These limited the generalizability of findings from this feasibility pilot project. However, the project purpose was to determine the feasibility of implementing VR-PAT in real-world clinical settings using a hand-off strategy. The preliminary data from this study provided a foundation for larger-scale research. Future studies should explore VR-PAT’s effectiveness across different demographics and medical conditions and assess long-term outcomes. Additionally, the lack of anonymous survey responses may introduce bias, as medical providers might have been reluctant to report negative experiences. Second, a limitation of the VR intervention used in this study was that the VR-PAT was designed for children aged 5 years or older, so younger children were excluded. It can be challenging to immerse young children in a developmentally appropriate VR. Furthermore, children 2–5 years old could have trouble in fully understanding and communicating their pain and anxiety scores. Our team recognizes the high anxiety needs of younger children and is exploring other innovative distraction methods (e.g., AR) for future research. Finally, because our previous publications^8,11^ described in detail the perception and experience of key stakeholders (patients, legal guardians, and nurses) in a relatively large randomized controlled trial of VR-PAT, this clinical implementation feasibility pilot study did not repeat those outcome measures. Readers could refer to those previous publications^8,11^ for our findings about these important outcome measures.

## Conclusion

In conclusion, this study demonstrates the potential for VR-PAT to become a standard tool for pediatric pain and anxiety management, showing positive feedback from both patients and providers. Collaboration between research and clinical units is essential to develop standardized guidelines for VR interventions, which could facilitate insurance reimbursement and wide adoption in clinical practice. Standardized VR pain and anxiety alleviation therapeutic guidelines will offer a stronger argument for insurance reimbursement for medical VR usage.

## Abbreviations Used

ED: emergency department
IRB: Institutional Review Board
QI: quality improvement
VR: virtual reality
VR-PAT: virtual reality pain alleviation therapeutic
