# Supplementary material for: Implementation of Virtual Reality Pain Alleviation Therapeutic into Routine Pediatric Clinical Care: Experiences and Perspectives of Stakeholders
Source: J Med Ext Real. 2024 Sep 26;1(1):179–85. doi: 10.1089/jmxr.2024.0018 (PMC11514146; doi:10.1089/jmxr.2024.0018)
Supplement: Supplementary Data S1 [file jmxr.2024.0018_supplementaryfile1.pdf]

Clinic Survey

Record ID	__________________________________
Clinical Location	◯ Burn Clinic<br>◯ ED<br>◯ Orthopedic (pin-pulling)
Age of Patient	__________________________________<br>(not birthdate)
Sex	◯ Male<br>◯ Female<br>◯ Undisclosed
Month of VR use	◯ January<br>◯ February<br>◯ March<br>◯ April<br>◯ May<br>◯ June<br>◯ July<br>◯ August<br>◯ September<br>◯ October<br>◯ November<br>◯ December
Year of VR use	◯ 2022<br>◯ 2023<br>◯ 2024
On a scale of 0-10, How much did you like playing the VR game during your procedure? (0=did not like at all, 10=liked it the most possible)	__________________________________<br>(for patient to answer)
Were there any technical issues with using the VR?	____<br>(for nurse to answer)
